# Coronary Artery Disease and Atherosclerosis in Other Vascular Districts: Epidemiology, Risk Factors and Atherosclerotic Plaque Features

**DOI:** 10.3390/life15081226

**Published:** 2025-08-03

**Authors:** Michele Russo, Filippo Luca Gurgoglione, Alessandro Russo, Riccardo Rinaldi, Laura Torlai Triglia, Matteo Foschi, Carlo Vigna, Rocco Vergallo, Rocco Antonio Montone, Umberto Benedetto, Giampaolo Niccoli, Marco Zimarino

**Affiliations:** 1 Department of Cardiology, SS. Annunziata Hospital, ASL2 Abruzzo, 66100 Chieti, Italy; m.zimarino@unich.it; 2 Department of Cardiology, F. Renzetti Hospital, ASL2 Abruzzo, 66034 Lanciano, Italy; 3 Department of Cardiology, S. Maria dei Battuti Hospital, AULSS 2 Veneto, 31015 Conegliano, Italy; 4 Department of Medicine, University of Parma, 43125 Parma, Italy; laura.torlait@gmail.com (L.T.T.); gniccoli73@hotmail.it (G.N.); 5 Cardiology Unit, Fondazione IRCCS Casa Sollievo della Sofferenza, 71013 San Giovanni Rotondo, Italy; alessandrorusso17@hotmail.it (A.R.); cvigna57@gmail.com (C.V.); 6 Cardiology Unit, Infermi Hospital, 47923 Rimini, Italy; rinaldiriccardo92@gmail.com; 7 Department of Biotechnological and Applied Clinical Sciences, University of L’Aquila, 67100 L’Aquila, Italy; mattfos89@gmail.com; 8 Interventional Cardiology Unit, Cardiothoracic and Vascular Department (DICATOV), IRCCS Ospedale Policlinico San Martino, 16132 Genova, Italy; roccovergallo@gmail.com; 9 Department of Internal Medicine and Medical Specialties (DIMI), Università di Genova, 16132 Genova, Italy; 10 Department of Cardiovascular and Pulmonary Sciences, Catholic University of the Sacred Heart, 00168 Rome, Italy; rocco.montone@gmail.com; 11 Department of Cardiovascular Sciences, Fondazione Policlinico Universitario A. Gemelli IRCCS, 00168 Rome, Italy; 12 Department of Cardiac Surgery, University “G. d’Annunzio”, 66100 Chieti, Italy; umberto.benedetto@asl2abruzzo.it; 13 Department of Neuroscience, Imaging and Clinical Sciences, “Gabriele d’Annunzio” University of Chieti-Pescara, 66100 Chieti, Italy

**Keywords:** coronary artery disease, atherosclerosis, polyvascular disease, risk factors, plaque features, plaque destabilization

## Abstract

Coronary artery disease (CAD) is the main cause of morbidity and death worldwide, and atherosclerosis represents the leading pathophysiological pathway responsible for CAD. Atherosclerotic process is a complex interplay of mechanisms and mediators resulting in plaque formation, progression and destabilization, the latter being the most frequent cause of acute cardiovascular events. Considering the systemic nature of atherosclerosis, polyvascular disease involvement is possible and has been described since 1960s. Accordingly, epidemiologic studies reported that concomitant CAD and atherosclerosis in other arterial beds like carotid arteries, lower limb arteries, mesenteric and renal circulation, and aorta, is frequent and related to increased chance of future cardiovascular events. Although risk factors, atherosclerotic plaque features and mechanisms of plaque destabilization are largely shared across different sites, many studies have reported some disparities among districts. Moreover, simultaneous polyvascular disease has been associated with increased likelihood of having particular plaque characteristics depending on the affected arterial level. In this comprehensive narrative review, we aim to discuss about epidemiology of concomitant CAD and atherosclerosis in other arterial beds, and to examine differences in risk factors, plaque features and mechanisms of plaque instability between CAD and other atherosclerotic locations. Finally, we review the studies observing differences on plaque features according to involved atherosclerotic sites, focusing on CAD.

## 1. Introduction

Atherosclerotic vascular disease (ASCVD), including ischemic heart disease (IHD), ischemic stroke and peripheral arterial disease (PAD) is one of the most important plague of modern health, causing altogether almost 13 million of deaths and 311 million of Disability-Adjusted Life Years (DALY) in 2022 worldwide [1]. IHD and stroke represent the first Level 3 global causes of death and DALYs among all diseases, respectively, and are expected to keep this record in 2050 as well, as shown in a recently published Global Burden of Disease 2021 forecasting analysis [2]. Atherosclerosis, representing the common pathophysiological substrate of ASCVD, is a complex and dynamic systemic disease enclosing a broad range of mechanisms and mediators [3]. In brief, atherogenesis starts with lipoprotein arterial subendothelial space deposition enhanced by endothelial cell dysfunction. This process is able to act as a stimulus for innate and adaptive immunity causing lipid phagocytosis by macrophages becoming “foam cells”. Subsequent intimal foam cell accumulation, cytokine and other substances release are able to amplify low-density lipoprotein (LDL) oxidation and local inflammatory response recruiting other inflammatory cells (i.e., neutrophils, eosinophils, T-helper lymphocytes, etc.). Vascular smooth muscle cells (VSMCs) activation and migration determine extracellular matrix component production with pathogenetic evolution from intimal xanthoma to pathological intimal thickening. Foam cells and VSMCs apoptosis and necrosis release lipid droplets in the intimal space and determine the formation of necrotic core. When necrotic core becomes enclosed by a fibrous cap, the transition to the fibroatheroma stage is complete. Inflammatory response amplification, neoangiogenesis, vascular remodelling and eventually calcification further contribute to the evolving atherosclerotic process. Lipid core increase, cholesterol crystal deposition in the intima, increased vascularization, enhanced inflammation and fibrous cap thinning may lead a plaque to become more prone to rupture, defined as “vulnerable”. Plaque rupture represents the most fearsome stage of atherosclerotic process, because fibrous cap disruption causes lipid and plaque debris release into the lumen causing local thrombosis and potentially vessel occlusion [3,4]. Plaque erosion represents a different mechanism of plaque destabilization and is related to the denudation of endothelium and tissue factor exposition causing leucocytes recruitment, platelet activation, fibrin generation and thrombosis. Calcified nodules represent the least frequent cause of plaque destabilization and are more frequently found in older people and female sex [3].

Atherosclerotic plaque formation and eventually destabilization are responsible for clinical manifestations of ASCVD, extending from stable ischemia (i.e., chronic coronary syndrome, chronic lower limb ischemia, chronic mesenteric ischemia) to acute clinical manifestations (i.e., myocardial infarction, ischemic stroke, acute mesenteric ischemia), with potentially fatal outcomes [5].

Considering the systemic nature of atherosclerosis as a pathogenetic entity, the coexistence of polyvascular atherosclerotic involvement in the same patient is common. In particular, evidence supporting this concept come from histological studies performed since 1960s which demonstrated that patients with coronary artery disease (CAD), the main cause of IHD, have frequently simultaneous carotid, iliac and aortic atherosclerosis. It has also been reported that the burden and severity of CAD was associated with atherosclerosis burden and severity in the other arterial districts [6,7]. Recent studies have also shown that atherosclerotic polyvascular disease involving more than one arterial bed in the same patient is associated with worse cardiovascular outcomes [8,9].

The advancement of atherosclerosis within single arterial districts and across different arterial beds is heterogeneous and often unpredictable with certainty. In addition, modifiable (i.e., hypertension, diabetes, hypercholesterolemia, smoking, etc.) and unmodifiable risk factors (i.e., sex, age, genetic substrate, etc.) may have different influence on its progression at different arterial sites. Histology and imaging studies have also shown that atherosclerotic plaque features and mechanisms of plaque destabilization may differ in prevalence across different vascular beds. Finally, increasing evidence indicated that plaque characteristics may diverge within the same patents according to the involved vascular sites [10,11]. The increasing imaging tools, both (1) non-invasive such as computed tomography angiography (CTA), allowing to study non-invasively atherosclerotic plaque burden, remodelling and features of vulnerability (i.e., napkin ring sign, microcalcification, etc.), and (2) invasive techniques like intravascular ultrasound (IVUS), showing optimal capability to assess plaque burden, plaque calcification and remodelling thanks to its high penetration depth, and optical coherence tomography (OCT), demonstrating the highest spatial resolution with excellent assessment of microstructures and features of vulnerability (i.e., thin cap fibroatheroma, macrophages), etc., have encouraged recent research on polyvascular plaque assessment in vivo [12].

Hence, in the present narrative review, we aim to discuss about epidemiology of concomitant CAD and atherosclerosis in districts other than coronary arteries, and to argue about differences in risk factors, plaque features and mechanisms of plaque instability between CAD and other atherosclerotic sites. Finally, we discuss, where available, about characteristics of plaques according to the simultaneous presence of atherosclerosis in multiple sites, focusing on CAD.

## 2. Coronary Artery Disease and Atherosclerotic Carotid Artery Disease

**Figure 1:**
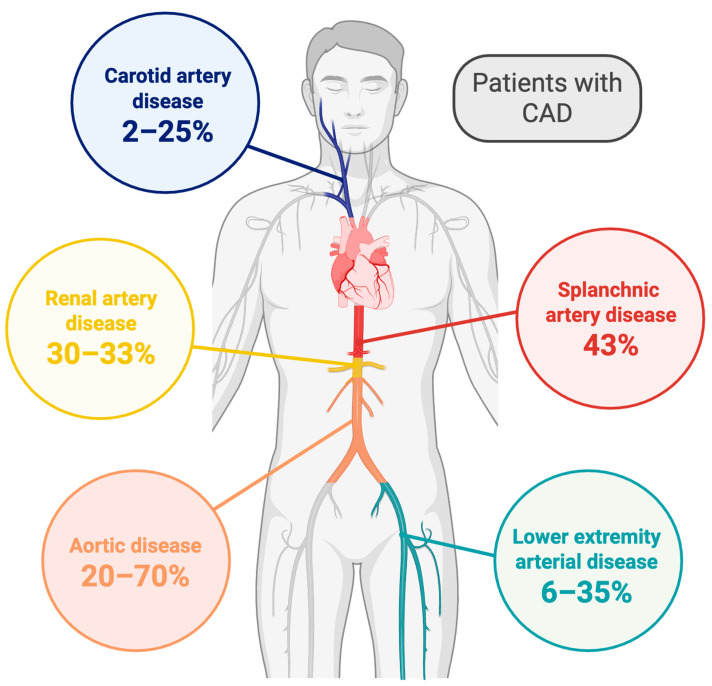
Prevalence of simultaneous CAD and other site arterial disease. CAD = coronary artery disease. This figure was created using BioRender.com.

Figure 1 graphically shows the prevalence of concomitant atherosclerosis in the coronary arteries and other arterial beds. Table 1 summarizes the main risk factors and the differences in plaque features and mechanisms of plaque instability between CAD and other districts, and shows main coronary artery plaque features in the presence of atherosclerotic disease in another level. Principles of medical therapy per district are also showed.

Ischemic stroke is the most frequent cause of stroke (more than 60% among all types) and has been estimated as the second cardiovascular cause of death after IHD, causing more than 3 million fatalities in 2022 [1]. Significant (≥50%) carotid artery stenosis is thought to be responsible for 15–20% of all ischemic stroke and transient ischemic attack [96]. The prevalence of carotid artery atherosclerosis is higher in the general population [97]. Carotid artery plaques and significant carotid artery stenosis have been estimated in 21.1% and 1.5% of global population aged 30–79 years old, respectively [97]. Carotid bulb at the bifurcation of the common carotid artery and the proximal internal carotid artery represent the most frequent sites of carotid artery atherosclerosis [98].

### 2.1. Prevalence of Concomitant CAD and Atherosclerotic Carotid Artery Disease

Multiple studies have reported that carotid artery disease and CAD are strictly correlated [7]. Significant carotid artery stenosis has been estimated in 2–25% of patients with CAD and 3–22% of patient undergoing coronary artery bypass grafting, with a frequency rising to 31% in patients with severe CAD (3-vessel CAD or left main stem disease) [74,99,100,101,102,103]. The prevalence of non-significant carotid artery stenosis in CAD patients is even higher, ranging from 43% to 96.9% across different studies [100,104,105,106]. Notably, the presence of carotid artery atherosclerosis in CAD patients has been associated with worse cardiovascular outcomes [107,108]. On the counterpart, significant CAD has been identified in 28–40% of patients undergoing carotid artery endarterectomy and in 20–41% of patients with stroke [109,110,111,112]. Additionally, coronary plaques of any grade were found in up to 79% of patients with fatal stroke having plaques in any segment of the extracranial and intracranial brain arteries [113].

### 2.2. Risk Factors

Older age, male sex, traditional (i.e., hypertension, diabetes mellitus, smoking, dyslipidemia, etc.) and other risk factors (i.e., environmental and household air pollution, hyperhomocysteinemia, hyperuricemia, etc.) have been associated with both coronary and carotid artery atherosclerosis [11,19,20]. In particular, hypertension, diabetes and current smoking have been reported to strongly correlate with atherosclerosis in the two districts; conversely, high levels of low-density lipoprotein cholesterol, although relevant, have been more weightily associated with coronary atherosclerosis [11,21,22]. Lower levels of high-density (HDL) cholesterol and atrial fibrillation have also been associated with carotid artery plaques [97,114]. Among other risk factors, in the recent Malmö Diet and Cancer Study including 26,681 patients, alcohol consumption, low physical activity and educational level were significantly correlated with CAD and ischemic stroke, although these factors were not statistically significantly associated with incidental carotid artery atherosclerosis [11].

### 2.3. Plaque Features and Mechanisms of Plaque Destabilization

**Figure 2:**
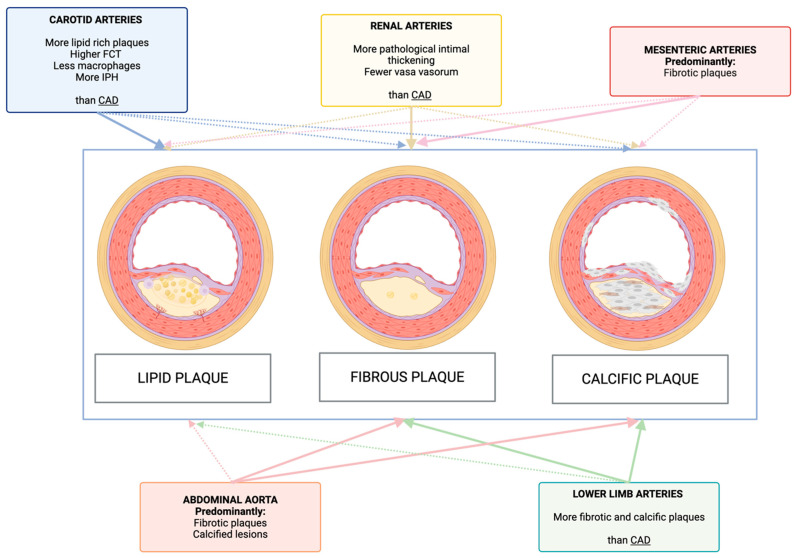
Plaque types according to different vascular districts. CAD = coronary artery disease; FCT = fibrous cap thickness; IPH = intraplaque hemorrhage. This figure was created using BioRender.com.

Histology and imaging findings have reported differences between carotid and coronary artery atherosclerosis, primarily due to higher flow rates and forces in the carotid circulation [36]. Carotid plaques were found to be more frequently lipid rich (70–90% of total) as compared to coronary plaques, while the incidence of calcifications was similar [37,38] (Figure 2). Fibrous cap was reported to be thicker and prevalence of macrophage infiltration in the fibrous cap lower in the carotid than in the coronary plaques [10]. Plaque rupture is the most frequent mechanism of plaque destabilization both in the carotid and coronary arteries, while plaque erosion is less common in the carotid (9.9% of ischemic stroke related to plaque destabilization) as compared to the coronary district (about one third of ACS related to plaque destabilization) [50,51,52,53]. Calcified nodules, the least common cause of plaque destabilization, were found to be more frequent in the carotid than coronary arteries (6–7% vs. 1–2%) [36].

Thrombus and plaque debris embolization is the main cause of cerebral ischemic events after carotid plaque destabilization, unlike coronary arteries where local artery occlusion is the primary determinant for the largest part of ACS. The detection of ulcerated plaques (cavities within the plaque), remnants of previous rupture and thrombus lysis/embolization, is much more common in the carotid arteries than coronary arteries; conversely, total occlusions are more frequent in the coronary arteries [36,38]. Intraplaque hemorrhage, an important cause of plaque progression and necrotic core expansion, have been more often identified in the carotid rather than in the coronary plaques because of carotid flow dynamics [39,40]. Finally, healed plaques, signs of previous plaque destabilization and healing, are frequently found in both narrow carotid and coronary arteries [36,41].

### 2.4. Characteristics of Plaques in the Case of Concomitant Atherosclerosis in the Two Arterial Beds

Many studies have reported differences in carotid artery atherosclerotic features in CAD patients. A large metanalysis by Bytyçi showed that the prevalence of calcifications and lipid-rich necrotic core in carotid plaques assessed by using magnetic resonance imaging (MRI) was higher in CAD patients than in controls. Interestingly, the prevalence of calcifications was greater and of lipid-rich necrotic core (LRNC) was lower in carotid plaques of patients with significant than non-significant CAD [7]. In another study, Mantella et al. found that intraplaque neovascularization in carotid arteries was associated with significant CAD and greater complexity of coronary lesions [115]. Conversely, Usman et al. did not found differences about large lipid core, ruptured plaques and intraplaque hemorrhage studied with MRI in the carotid arteries of patients with and without CAD [116].

The correlation between coronary and carotid plaque features has been assessed in many studies by using different imaging modalities. A study by Zhao et al. [55] correlated carotid plaque phenotype evaluated by MRI and coronary plaque features studied by CTA. The authors found that coronary calcified plaque score had the highest predictive value for carotid calcification; moreover, calcified coronary plaque was the strongest predictor of LRNC in carotid plaques. They also reported that the mixed coronary plaque score was significantly correlated with carotid IPH [55]. In another study, Zhang et al. stated that noncalcified plaque score in coronary arteries can predict the presence of noncalcified plaque in the carotid arteries; moreover, the calcified coronary plaque score predicted the presence of mixed and calcified plaque in the carotid arteries [56]. Other CT reports confirmed the association between calcified coronary and carotid plaques [57,58]. Lee et al. also described a relationship between irregular plaque surface assessed by carotid ultrasonography and coronary artery calcium score (CACs) [59]. In a virtual histology IVUS (VH-IVUS) study, a moderate correlation was found between necrotic core (r = 0.46, *p* < 0.01), fibrotic tissue (r = 0.42, *p* < 0.01), fibro-fatty tissue (r = 0.37, *p* < 0.01), and dense calcium tissue (r = 0.56, *p* < 0.01) in coronary and carotid lesions [37]. Saito et al. also revealed that morphologically unstable plaques of the carotid artery predicted unstable forms of coronary obstruction with a sensitivity, specificity, predictive power and likelihood ratio of 68%, 85%, 72% and 4.5, respectively [60]. In this regard, many studies reported signs of panvascular inflammation in both coronary and carotid arteries [61,62,63]. An intracoronary optical coherence tomography (OCT) study assessing mechanisms of plaque destabilization in the coronary arteries reported that patients with coronary plaque erosion causing ACS had the lowest prevalence of heterogenous and calcified carotid plaques [64]. These data have been confirmed by Weng et al., who demonstrated that the prevalence of carotid vulnerable plaque features like plaque surface irregularity, heterogeneous plaque, and calcifications was lower in patients with coronary plaque erosion than plaque rupture [65]. Finally, Liu et al. described that vulnerable coronary plaques evaluated by OCT were significantly associated with irregular (OR = 4.819; 95% CI: 1.106–22.867) and hypoechoic (OR = 9.632; 95% CI: 2.138–43.384) carotid plaques at ultrasound examination [66]. These data suggest that atherosclerotic phenotype is similarly expressed in both coronary and carotid arteries.

### 2.5. Therapeutic Implications

Considering the role of risk factors in determining both forms of ASCVDs, lifestyle modifications (i.e., stop smoking, physical activity, weight loss, optimal diet, etc.) and drug intervention are essential to prevent atherosclerotic disease progression in both districts [75,76]. European Society for Vascular Surgery 2023 Clinical Practice Guidelines recommend anti-hypertensive and glycemic control treatment in patients with hypertension and diabetes, respectively, and asymptomatic and symptomatic carotid stenosis. About lipid lowering therapies, statins are recommended in asymptomatic and symptomatic carotid artery stenosis [75]. In this regard, statins and proprotein convertase subtilisin/kexin type 9 (PCSK9) inhibitors have been reported to promote plaque stabilization in both coronary and carotid plaques [77,78,79,80]. About the role of aspirin, European Society for Vascular Surgery 2023 guidelines indicates that low dose aspirin (75–325 mg daily) should be considered in patients with asymptomatic carotid artery stenosis >50%, mainly for the prevention of late myocardial infarction and other cardiovascular events [75]. Recent ESC 2024 guidelines on PAD give a class IIa recommendation for the use of antiplatelet therapy in patients with asymptomatic carotid artery stenosis >50% if bleeding risk is low [74]. A recent study by Dzaye suggested that quantification of subclinical carotid atherosclerosis based on carotid plaque score by using carotid ultrasound can help in the decision of introducing aspirin therapy [117]. This concept reflects the one reported in coronary arteries about drug modulation according to atherosclerosis severity, complexity and the therapeutic strategy [118]. Finally, the use of anti-inflammatory drugs like colchicine has shown efficacy in preventing both ischemic coronary events and recurrent strokes [81,82,83]. Future studies will help to refine personalized and targeted therapies in patients with carotid artery atherosclerosis and CAD.

## 3. Coronary Artery Disease and Atherosclerotic Lower Extremity Arterial Disease

Lower extremity arterial disease (LEAD) is characterized by the presence of atherosclerotic lesions in the arteries supplying the legs and is the third most common manifestation of atherosclerotic cardiovascular disease [119]. LEAD can involve both proximal (femoral and popliteal arteries) and distal districts (anterior tibial, posterior tibial, peroneal, and dorsalis pedis arteries) [54].

### 3.1. Prevalence of Concomitant CAD and LEAD

A well-established association exists between LEAD and CAD, with both conditions coexisting in 6–35% of patients [120,121]. The wide variability in this range may be attributed to the frequent underdiagnosis of LEAD, which often progresses with a clinically silent course [122]. The presence of LEAD is associated with a 70% increased risk of developing CAD, with a higher incidence of multivessel CAD and left main involvement [123]. Furthermore, LEAD nearly doubles the rates of percutaneous coronary intervention failure [124,125] as well as all-cause and cardiovascular mortality [126].

### 3.2. Risk Factors

Although both CAD and PAD are manifestations of atherosclerotic disease, they exhibit some difference in risk factors. Smoking is a major risk factor for atherosclerotic disease, with a stronger relationship with PAD than with CAD [23]. The ARIC study demonstrated that heavy smoking (≥1 pack/day) was associated with nearly a 2.3-fold higher risk of LEAD compared to CAD, and that prolonged smoking duration (≥35 years) conferred a 2.4-fold higher risk of LEAD. Notably, the increased risk of PAD persists for up to 30 years after smoking cessation [24]. Diabetes is more strongly associated with LEAD than with other manifestations of atherosclerosis, particularly in men, with a relative risk for LEAD versus CAD of 44% [11]. The pathogenesis of LEAD in diabetes is likely driven by both hyperglycemia-induced microvascular and macrovascular damage [25]. Additionally, the contribution of hypertension is slightly more pronounced in LEAD compared to CAD [11]. Patients with LEAD often exhibit a distinct lipid profile, characterized by elevated triglyceride levels and reduced high-density lipoprotein cholesterol [26]. The risk factor profile differs based on the site of the atherosclerotic lesion. Femoral and popliteal disease is strongly associated with cigarette smoking and dyslipidemia, whereas distal district is more commonly linked to diabetes and advanced age [27]. The correlation between genetics and risk factors (i.e., diabetes, smoking, etc.) in the pathogenesis of PAD have also been reported [127]. Finally, lower estimated glomerular filtration rate was found to significantly correlate with increased risk of PAD [128].

### 3.3. Plaque Features and Mechanisms of Plaque Destabilization

Differences in risk factors, intrinsic vessel features (such as diameter and shear stress) [129] and variations in gene expression [130], contribute to the distinct composition of plaques between CAD and LEAD. Histological and IVUS studies have shown a predominance of fibrotic and calcified plaques in femoropopliteal arteries, with only 25% of lesions containing a lipid-rich necrotic core [42,43,44] (Figure 2). Additionally, LEAD presents distinctive mechanisms of plaque destabilization, with a significant role of calcified nodules, chronic luminal thrombus, even in the absence of significant atherosclerotic disease, and embolic events [23,54].

### 3.4. Characteristics of Plaques in the Case of Concomitant Atherosclerosis in the Two Arterial Beds

Of interest, the concomitant presence of LEAD might influence the CAD phenotype, as shown in imaging studies. A small computed tomography coronary angiography study revealed a higher number of mixed coronary plaques in patients with lower ankle-brachial index, which is diagnostic for LEAD [67]. Hussein and colleagues conducted a large patient-level pooled analysis of 7 IVUS clinical trials addressing CAD features through serial IVUS examinations. Patients with LEAD exhibited a greater percent atheroma volume (40.4 ± 9.2 vs. 38.5 ± 9.1, *p* = 0.002) and a higher prevalence of calcified plaques compared to those without PAD. IVUS follow-up revealed a greater progression of percent atheroma volume in the LEAD group both in the unmatched (±0.58 ± 0.38% vs. ±0.23 ± 0.3%, *p* = 0.009) and matched populations (±0.54 ± 0.38% vs. ±0.23 ± 0.33%, *p* = 0.03), as well lower rates of plaque regression (15.3% vs. 22.3%, *p* = 0.02) [68].

Bryniarski and colleagues investigated both culprit and non-culprit coronary lesions using OCT among 102 patients with CAD, 67.6% of whom had ACS. Patients with LEAD had a higher prevalence of vulnerable culprit coronary lesions, as evidenced by a greater maximum lipid arc (257.4° ± 81.0 vs. 206.3° ± 65.1, *p* = 0.002), increased macrophage accumulation (70.8% vs. 48.5%, *p* = 0.034) and cholesterol crystals (32.4% vs. 10.3%, *p* = 0.006), and more calcifications (79.4% vs. 58.8%, *p* = 0.039) compared to those without LEAD. Similarly, non-culprit lesions in the LEAD group were longer (40.4 mm ± 9.2 vs. 38.5 mm ± 9.1, *p* < 0.001) and with higher rates of macrophage accumulation (63.3% vs. 38.3%, *p* = 0.025), cholesterol crystals (36.7% vs. 16.7%, *p* = 0.034), and calcifications (76.7% vs. 55.0%, *p* = 0.046) compared to those without LEAD [69].

### 3.5. Therapeutic Implications

Altogether, these findings suggest that patients with concomitant CAD and LEAD have more aggressive atherosclerotic disease, with greater pancoronary vulnerability. Therefore, aggressive risk management may offer prognostic benefits [84,85]. In recent years, evolocumab has demonstrated superiority over placebo in reducing the composite endpoint of cardiovascular death, myocardial infarction, ischemic stroke, hospital admission for unstable angina, or coronary revascularization, both in patients with LEAD and CAD, with hazard ratios [HRs] of 0.79 and 0.85, respectively [88,89]. Similarly, the Cardiovascular Outcomes for People Using Anticoagulation Strategies (COMPASS) trial reported a reduced incidence of major adverse cardiovascular events with rivaroxaban, in addition to aspirin, compared to aspirin alone, in both patients with LEAD and CAD, with HRs of 0.71 and 0.74, respectively [86,87].

## 4. Coronary Artery Disease and Mesenteric Artery Atherosclerosis

Atherosclerotic disease is the main cause of chronic gastrointestinal ischemia, characterized by an inadequate intestinal blood flow achievement after meals due to an unbalance between oxygen and metabolite supply and demand. The superior mesenteric artery, celiac artery and less frequently the inferior mesenteric artery are the vessels more frequently involved [131]. The prevalence of clinically evident chronic abdominal ischemia is significantly lower than other ASCVDs, with an estimated prevalence of only 30 cases per 100,000 individuals [132,133]. The higher presence of collateral vessels in the gastrointestinal tract explains the discrepancy between the prevalence of visceral atherosclerosis and symptomatic chronic gastrointestinal ischemia. The predominance of stenosis in the mesenteric district differs across clinical studies and examined population. Wilson et al. reported that 17% of elderly adults exhibited significant occlusive disease in the superior mesenteric artery or celiac artery [131]. In another larger study involving 713 patients, a similar prevalence of stenosis or occlusion of at least one of the gastrointestinal arteries was found [134]. In this regard a strong correlation between gastrointestinal arterial stenosis and ageing has been reported [135]. Atherosclerotic plaques tend to occur at the ostia of the vessels and in the first 2–3 cm of the main trunks. The stenosis of superior mesenteric artery typically occurs in its proximal segment, with the iliocoecal region showing the highest risk of ischemia [136].

### 4.1. Prevalence of Concomitant CAD and Gastrointestinal Artery Diseases

Some studies investigated the incidence of mesenteric arteries stenosis in CAD patients. In a cohort of patients undergoing surgery for chronic gastrointestinal ischemia the incidence of CAD was about 33% [137]. In a large meta-analysis involving more than 18,000 patients with chronic gastrointestinal ischemia undergoing visceral artery revascularization (both surgical and percutaneous), CAD was present in about 37% of patients [28]. In another report including 103 patients with CAD undergoing coronary and mesenteric angiogram, about 43% had mesenteric artery stenosis, with involvement of the superior mesenteric artery, celiac artery and inferior mesenteric artery in 39%, 22% and 15% of cases, respectively [138].

### 4.2. Risk Factors

Female sex is more frequently interested by chronic gastrointestinal ischemia than males, as opposed to the higher prevalence of males in CAD [14]. The underlying reason is unclear, although it has been supposed that a more acute angle between mesenteric vessels and the aorta may predispose females to develop mesenteric artery stenosis [15,16]. Along with sex, age, smoking, hypertension and dyslipidemia are the most important risk factors for mesenteric atherosclerosis [28]. The prevalence of hypercholesterolemia and diabetes in patients with chronic splanchnic syndrome is lower as compared to CAD. A reason may be that reduced macronutrients intake with consequent lower body weight may limit the impact of these risk factors in chronic gastrointestinal ischemia [29,30]. Superior mesenteric artery calcification has been significantly associated with an increased risk of both total and cardiovascular mortality [31].

### 4.3. Plaque Features and Mechanisms of Plaque Destabilization

In a rabbit atherosclerotic model, mesenteric artery lesions were mainly fibrotic while the celiac artery showed foam cells and/or lipid droplets within fibrous lesions [45] (Figure 2). In a histology study, more severe atherosclerosis was found in the superior mesenteric artery rather than inferior mesenteric artery, with the former having more vulnerable plaque features enclosing a thin fibrous cap with large number of macrophages underneath the cap and at the plaque shoulder than the latter [46].

Acute mesenteric ischemia may occur from either embolic events or local thrombosis at atherosclerotic lesion sites. Arterial emboli are more frequently cause of acute mesenteric ischemia than in situ thrombosis (embolic/thrombotic ratio: 1.4 to 1.0), while the latter is responsible for 42% of the cases [139,140]. Usually, thrombotic occlusion appears as a clot superimposed on a heavily calcified occlusive lesion at the origin of the superior mesenteric artery, whereas embolic occlusion often manifests as an oval-shaped filling defect surrounded by contrast in a non-calcified arterial segment located in the middle and distal part of the superior mesenteric artery [141]. Cases of plaque rupture has been described at thrombotic superior mesenteric artery sites [140] although systematic studies assessing the prevalence of different pathobiology of plaque destabilization and local thrombosis in the superior mesenteric artery are lacking.

### 4.4. Characteristics of Plaques in the Case of Concomitant Atherosclerosis in the Two Arterial Beds

Few studies have evaluated the association between coronary and mesenteric atherosclerotic plaque features. In this regard, Lin et al., showed by using CTA, that superior mesenteric artery calcification increased by 1.74-fold (95% CI: 1.14 to 2.67) the risk of coronary artery calcifications [31]. Future research about the association between mesenteric and coronary plaque features is warranted.

### 4.5. Therapeutic Implications

Reports about pharmacological approaches for secondary prevention in patients with asymptomatic mesenteric artery stenosis are currently lacking. Considering the systemic nature of atherosclerotic disease, current guidelines suggest that control of risk factors and pharmacological treatments where indicated (i.e., lipid lowering therapies, anti-hypertensive drugs, hypoglycemic therapies) are advised [74,92]. In a study by Alnahhal et al. including 278 patients undergoing endovascular intervention for chronic mesenteric ischemia, preoperative high-intensity statins were associated with lower primary lumen patency loss as opposed to none or less powerful statins (HR, 0.30; 95% CI: 0.11–0.72; *p* = 0.014) [93]. In symptomatic chronic mesenteric ischemia, endovascular or open surgery remain the mainstay for vessel revascularization [74].

## 5. Coronary Artery Disease and Renal Artery Atherosclerosis

Renal artery stenosis is the most common secondary cause of hypertension and it is predominantly caused by atherosclerosis. Renal artery stenosis typically involves the proximal segment of the renal artery, generally due to ingrowth of aortic plaques into the renal artery ostium. In advanced cases, segmental and diffuse intrarenal atherosclerosis may also be observed, particularly in patients with ischemic nephropathy [142]. Renal artery stenosis is predominately an age-dependent pathology. The prevalence is 10–15% in hypertensive patients over the age of 50 years and increases to 50–60% in elderly patients with hypertension, atherosclerotic coronary and peripheral artery disease, and renal dysfunction [143]. Stenosis in the renal artery is a strong indicator of increased mortality and a risk factor for cardiovascular diseases, myocardial infarction, ischemic stroke and heart failure [94].

### 5.1. Prevalence of Concomitant CAD and Renal Artery Stenosis

The association between renal artery stenosis and CAD is well known. In patients with CAD, the incidence of renal artery stenosis is about 30–33%, of which 7–15% patients have a stenosis greater than 50%. Bilateral stenosis can be found in 4–11% of patients [144,145]. The prevalence of renal artery stenosis is estimated in more than 40% of patients with LEAD and in more than 50% of patients with polyvascular atherosclerosis [146].

### 5.2. Risk Factors

Age, female sex, hypertension, presence of generalized atherosclerosis, multivessel CAD, diabetes, elevated creatinine and LDL cholesterol levels have been all identified as independent risk factors for renal artery stenosis in multivariate analysis [17]. The development of renal atrophy and/or decline in measured glomerular filtration rate (GFR) occurred in nearly 50% of patients within 3 years [147].

### 5.3. Plaque Features and Mechanisms of Plaque Destabilization

About atherosclerotic plaque features, Matsuo et al. [47], by using VH-IVUS, showed that the largest plaque type in renal artery stenosis was pathological intimal thickening (60%), followed by fibroatheroma (22%)-of which 16% were thin cap fibroatheroma and 6% thick-cap fibroatheroma-and fibrocalcific plaque (18%). Coronary arteries had higher prevalence of fibroatheroma (50% vs. 22%, *p* < 0.001) and lower prevalence of pathological intimal thickening (18% vs. 60%, *p* < 0.001) as compared to renal artery lesions, respectively [47] (Figure 2). In another VH-IVUS comparing renal artery and coronary plaque features, mean %fibrous tissue area (61% vs. 57%), mean % fibro-fatty area (21% vs. 18%) and mean % necrotic core area (11% vs. 14%) were similar between renal and coronary arteries. Conversely, mean % dense calcium area in renal arteries was lower than coronary arteries (6% vs. 11%, *p* = 0.003). The authors also reported that adaptive vessel enlargement was more strongly predicted by dense calcium in renal artery stenosis, while necrotic core is much important in CAD. It has been hypothesized that blood flow hemodynamics (renal blood flow is known to be markedly higher than coronary blood flow) may potentially explain these differences [148]. Histology studies have also showed lower density of vasa vasorum, implied in plaque growth and IPH, in renal arteries than coronary arteries [48].

Hypoperfusion is considered the predominant mechanism of ischemia in renal artery disease, although plaque rupture has been found in renal arteries, and atherothrombosis and cholesterol crystal embolization have reported to play a potential role in acute renal ischemia [47,149,150].

### 5.4. Characteristics of Plaques in the Case of Concomitant Atherosclerosis in the Two Arterial Beds

As previously reported, renal artery stenosis is a major cause of chronic kidney disease (CKD). CKD patients are more likely to have elevated serum phosphate levels due secondary hyperparathyroidism with increased risk of plaque calcification. CKD is also associated with a higher degree of systemic inflammation which is known to be an important trigger for atherosclerosis. Several studies demonstrated that the presence of inflammation within vessel wall induces atherosclerotic calcification [151].

Many reports have assessed coronary atherosclerotic features in patient with CKD. A sub-analysis of the PROSPECT (Providing Regional Observations to Study Predictors of Events in the Coronary Tree) trial showed, by using VH-IVUS, a greater prevalence of necrotic core and dense calcium at non-culprit coronary sites in non-hemodialysis CKD subjects, along with greater plaque burden and smaller minimal luminal area. At multivariate analysis, non-hemodialysis CKD was an independent predictor of percent necrotic core volume [70]. In an OCT study, the presence of CKD was associated with greater lipid atheroma burden and higher frequency of calcification. In addition, cholesterol crystals and plaque disruption were more frequently observed at non-culprit coronary lesions in patients with non-hemodialysis CKD, whereas no significant differences in fibrous cap thickness and prevalence of macrophage, microchannels and thin cap fibroatheroma were found [71]. Finally, Chin et al., analyzing OCT coronary plaque features in patients in chronic hemodialysis, showed that these patients had greater calcification arc at culprit and non-culprit lesions, and higher prevalence of intimal thin calcium and calcified nodules [72]. Coronary microvascular dysfunction has also been described in CKD patients, and it has been considered is as an important factor predicting mortality and adverse outcomes in these patients [152].

### 5.5. Therapeutic Implications

The previous findings suggest that worsening renal function, particularly in the cases requiring hemodialysis, is associated with more severe coronary atherosclerosis, and, in particular, to increased calcification and smaller lipid components [153]. The response of calcified atheroma to lowering LDL-C has been investigated by an IVUS imaging study. Interestingly, in this analysis calcific coronary atheroma continued to progress even under strict LDL-C control thus suggesting that CKD patients, having more extensive plaque calcifications, might have lower response to lipid lowering therapies [154].

As indicated for the other districts, the control of risk factors, in particular of hypertension, through lifestyle modification and drugs is the mainstay of medical management of renal artery stenosis. Percutaneous angioplasty and stenting, and surgery, are the therapeutic options for renal artery revascularization [94].

## 6. Coronary Artery Disease and Aortic Atherosclerosis

Aortic atherosclerosis is characterized by structural abnormalities of the aortic wall, including intimal thickening, atheromatous plaque formation, calcification, and aneurysmal dilatation, and represents a clinically relevant manifestation of systemic atherosclerotic disease. Thoracic aortic plaques and aneurysms are frequently detected incidentally on imaging; thoracic aortic aneurysms are defined as a permanent dilatation of the thoracic aorta exceeding 1.5 times their expected diameter, often remaining asymptomatic until reaching critical dimensions or showing rapid growth. Abdominal aortic aneurysms (AAAs) represent a more advanced stage of aortic atherosclerosis, and are defined by a permanent dilatation >3.0 cm in diameter [155].

### 6.1. Prevalence of Concomitant CAD and Aortic Disease

A consistent association exists between aortic atherosclerosis and CAD, which frequently coexist, especially in individuals with extensive atherosclerotic burden. In the REACH registry, 18% of patients with established atherosclerotic disease had both CAD and PAD, encompassing subclinical aortic involvement [156]. Among patients undergoing coronary angiography, incidental aortic plaques have been identified in 20% to 60% of cases, depending on imaging modality and study population. AAA is comorbid with obstructive CAD in up to 70% of cases, particularly in those undergoing surgical intervention [157,158].

### 6.2. Risk Factors

CAD and aortic atherosclerosis share common cardiovascular risk factors such as older age, male sex, smoking, hypertension, dyslipidemia, and systemic inflammation [18]. Notably, hypertension, the heaviest risk factor causing cardiovascular events, is strongly associated with AAA, likely due to its role in mechanical wall stress and remodelling [32,159]. Conversely, despite being a potent risk factor for CAD, diabetes appears to be inversely related to AAA formation, possibly due to glycation-mediated stabilization of extracellular matrix components [33]. Dyslipidemia and chronic inflammation are central drivers of vascular injury and atherosclerotic progression in both conditions [34,35,160,161].

### 6.3. Plaque Features and Mechanisms of Plaque Destabilization

CAD and aortic atherosclerosis have shown different pathophysiological features [5,162]. CAD typically affects medium-sized muscular arteries and is characterized by fibro atheromatous plaques containing lipid-rich necrotic cores and thin fibrous caps, making them prone to rupture and thrombosis [163]. Conversely, aortic atherosclerosis involves both intimal lipid accumulation and medial degeneration, with plaques, particularly in the abdominal aorta, being more fibrotic and heavily calcified [49] (Figure 2). Medial calcification (i.e., Monckeberg-type sclerosis), plaque ulceration, and intraluminal thrombus formation are common. Accordingly, unlike the rupture-prone coronary plaques, aortic plaques are generally more stable and are more often implicated in embolic rather than thrombotic events, especially during endovascular procedures [164]. The pathogenesis of aortic aneurysmal disease extends beyond atherosclerosis and involves complex remodelling processes driven by chronic inflammation, matrix metalloproteinase overexpression, reduced activity of tissue inhibitors of metalloproteinases, smooth muscle cell apoptosis, and degradation of the extracellular matrix. These processes result in progressive weakening of the aortic wall. Intraluminal thrombus, frequently present in AAA and occasionally in thoracic aortic aneurysms, exerts a dual effect: it may provide mechanical insulation from pulsatile stress, yet contributes to localized proteolysis and mural deterioration at the thrombus–wall interface [165].

### 6.4. Characteristics of Plaques in the Case of Concomitant Atherosclerosis in the Two Arterial Beds

Imaging studies investigating coronary plaque phenotype in patients with aortic atherosclerosis remain limited. A study by Acarturk et al. performed by transoesophageal echocardiogram showed that the largest part of CAD patients had more advanced aortic plaques, and that there was a correlation between aortic plaque severity and coronary artery disease [166]. In a study by Momiyama et al. assessing aortic plaques by using MRI, the authors reported that in patients with complex coronary lesions, complex aortic plaques in thoracic and abdominal aorta were more prevalent [167].

A recent study by Yuki et al. [73] described that patients having protruding plaques in aorta studied by CTA had higher coronary plaque vulnerable features assessed by OCT. In particular, these patients had higher prevalence of thin cap fibroatheroma (45.7% vs. 28.0%), lipid-rich plaques (88.7% vs. 80.7%), macrophages (79.0% vs. 68.5%), layered plaques (62.9% vs. 49.7%) and plaque rupture (51.6% vs. 25.9%) than patients without protruding aortic plaques [73].

### 6.5. Therapeutic Implications

From a therapeutic perspective, CAD and aortic atherosclerosis are managed with overlapping pharmacologic strategies targeting lipid reduction, blood pressure control, and platelet inhibition [168]. Lifestyle interventions, including smoking cessation, weight control, and regular physical activity, are universally recommended [169]. Statins have consistently demonstrated efficacy in reducing progression of both coronary and aortic plaque burden and may slightly decelerate AAA expansion, although their ability to reduce rupture risk remains uncertain [95]. Antiplatelet therapy is standard in the secondary prevention of CAD and PAD. 2024 ESC Guidelines for the management of PAD and aortic disease currently recommend antiplatelet therapy as secondary prevention for embolic events related to aortic atherosclerosis, and as primary prevention in patients with complex/severe aortic plaques [74]. Recent clinical trials have investigated the benefit of intensified pharmacologic therapy in systemic atherosclerosis. The COMPASS trial, for example, demonstrated that the addition of low-dose rivaroxaban (2.5 mg twice daily) to aspirin reduced major adverse cardiovascular events in patients with stable atherosclerotic disease, including CAD and PAD [170]. Whether similar benefits extend to patients with coexisting aortic disease remains to be elucidated. Surgical and endovascular interventions remain the mainstay of treatment for aneurysmal aortic disease, particularly when aneurysms exceed recommended size thresholds or demonstrate rapid growth.

## 7. Clinical Perspectives and Future Directions

The strict correlation existing between coronary and non-coronary atherosclerosis in terms of prevalence and atherosclerotic plaque features has important clinical implications. At first, the identification of atherosclerotic disease in districts other than coronary arteries should be considered to enhance cardiological risk stratification and secondary pharmacological prevention. As previously seen, the identification of carotid stenosis or PAD significantly increase the likelihood of obstructive CAD and the risk of future cardiovascular events [107,108,109,123,126]. In this regard, the identification of non-coronary atherosclerosis in patients with symptoms of unclear nature but potentially of cardiac origin should enhance a more careful diagnostic evaluation to exclude the presence of CAD. Moreover, 2024 ESC guidelines for the management of chronic coronary syndromes (CCS) also recommend considering carotid ultrasound as an alternative when CACS is unavailable or not feasible for atherosclerotic disease detection in asymptomatic patients, with the aim to improve risk classification and treatment decision thresholds [171]. The high prevalence of carotid artery stenosis and subsequent risk of stroke in patients undergoing CABG explain the indication of Society for Vascular Surgery to carotid artery screening before undergoing CABG for high-risk patients aged >55 years with cardiovascular risk [172]. From a therapeutic perspective, along with statins, the use of more recent lipid-lowering drugs like iPCSK9 has shown important positive results in multiple vascular bed atherosclerosis (coronary, carotid and lower extremity artery disease) showing benefits in plaque stabilization [80,88,89]. Considering that the presence of polyvascular disease has been associated with higher rate of plaque progression, the identification of atherosclerosis in multiple beds in patients with suboptimal LDL level controls should further enhance to consider a more aggressive lipid-lowering control by using these drugs, in particular if vulnerability plaque features have been found in one district [66,68]. Research about the use of other lipid-lowering drugs like bempedoic acid in non-coronary districts is ongoing.

Among anti-inflammatory drugs, colchicine has shown benefits in cardiovascular, cerebrovascular disease and PAD [90]. Accordingly, 2024 ESC guidelines for the management of CCS recently suggested to consider the use of colchicine in CCS patients with obstructive CAD to reduce the risk of myocardial infarction, stroke and the need for revascularization [171]. Similarly, canakinumab, demonstrating to have positive prognostic results in patients with prior myocardial infarction, has shown clinical benefit in PAD patients in preliminary results [91,173]. Future studies about the use of anti-inflammatory drugs in polyvascular disease patients are warranted. Finally, in a sub analysis of the COMPASS trial studying antithrombotic regimen with cardioaspirin + rivaroxaban, patients with polyvascular disease had better results in event risk reduction suggesting that stronger preventive antithrombotic treatments should be considered in polyvascular disease patients [86].

As previously discussed, plaque features in one district may correlate with the presence of atherosclerosis and plaque characteristics in another vascular site. This data may have implications in the case of interventional or surgical strategies to treat obstructive atherosclerosis in one district, because it may allow to provide information for pre-operative risk, and to predict peri-operative outcomes and possible complications. For example, calcified coronary lesions, more frequently found in patients with CKD, LEAD or in case of calcifications in carotid plaques, are associated with lower success PCI than non-calcified plaques [174]. Similarly, higher coronary plaque lipid burden, as more frequently seen in patients with LEAD, CKD and those with protruding aortic plaques, is associated with lower mean TIMI-flow grade after PCI, probably related to distal microembolization [175]. Currently, although panvascular vulnerability and higher risk of coronary events in patients with non-coronary vulnerable plaque features have been reported, benefits of preventive interventional or surgical treatment of coronary plaques when vulnerable plaque features are found in other districts have not been studied yet. Finally, intravascular imaging studies about mechanisms of plaque destabilization in mesenteric, renal and aortic districts are lacking, as well as the correlation between plaque features in some districts (i.e., mesenteric) and coronary plaques. Future studies may offer important information about this topic with the aim to further stratify and adapt personalized treatments in patients with polyvascular disease and vulnerable plaque features in one district. Recent research reported that specific circulating biomarkers are associated with atherosclerosis in distinct arterial districts and polyvascular disease. For example, C-reactive protein and β2-microglobulin have been associated with PAD; cystatin C and decreased cathepsin S/cystatin C ratio have been linked to PAD and polyvascular disease; triglyceride-glucose (TyG) index to carotid plaque instability; C-reactive protein and serum total bilirubin have been related with polyvascular disease [176,177,178,179,180]. However, routinely use of circulating biomarkers for preventive screening of district and polyvascular atherosclerosis is currently unadopted in clinical practice. Forthcoming studies on large population will clarify the impact and benefit of implementation of these biomarkers in real-life patient care for the assessment of subclinical polyvascular atherosclerosis.

## 8. Conclusions

In patients with CAD, atherosclerotic involvement of other arterial districts is common and related to increased risk of cardiovascular events. Although risk factors are largely shared, their impact in favouring atherosclerosis progression at different arterial levels is variable. Similarly, atherosclerotic plaque features and mechanisms of plaque destabilization change with atherosclerosis sites, reflecting mainly local hemodynamics and the role of specific risk factors. Patients having multilevel atherosclerosis may develop particular plaque features according to the interested site. Hence, the identification of “polyvascular disease patient” and the definition of its atherosclerotic profile may offer important clinical information with the aim to enhance a more aggressive lifestyle change and control of risk factors, and to tailor a personalized therapeutic approach to avoid abrupt clinical consequences of ASCVDs.

## Figures and Tables

**Table 1:** Differences in risk factors, atherosclerotic plaque features and mechanisms of plaque instability between coronary artery disease and atherosclerosis in carotid, lower limb, mesenteric, renal arteries and aorta. CAD features according to different arterial district involvement. Principles of medical therapy per district are also shown.

Atherosclerotic District	Carotid Artery Disease	Lower Limb Artery Disease	Mesenteric Artery Disease	Renal Artery Disease	Aortic Disease
Most affected sex	Male [11]	Male = Female [13]	Female [14,15,16]	Female [17]	Male [18]
Main risk factors	Age, hypertension, smoking and diabetes. Lower weight of dyslipidemia than CAD [11,19,20,21,22]	Age. Hypertension, smoking and diabetes greater weight than CAD. Particular lipid profile (more triglycerides and lower HDL) [11,23,24,25,26,27]	Age, smoking and hypertension. Lower weight of hypercholesterolemia and diabetes than CAD [28,29,30,31]	Age, hypertension, diabetes and dyslipidemia (high LDL) [17]	Age, hypertension, smoking and dyslipidemia. Diabetes mellitus inversely related to abdominal aortic aneurysms [18,32,33,34,35]
Main plaque features (vs. CAD)	More lipid rich plaques, higher FCT, less macrophages and more IPH. Similar calcifications and healed lesions [10,36,37,38,39,40,41]	More fibrotic and calcified lesions in femoropopliteal arteries [42,43,44]	No dedicated studies. Mesenteric artery lesions were mainly fibrotic in a rabbit model. More severe atherosclerosis and vulnerable plaques in SMA than IMA [45,46]	More PIT, lower prevalence of fibroatheroma and lower density of vasa vasorum [47,48]	No dedicated studies. More fibrotic and heavily calcified plaque in the abdominal aorta [49]
Mechanisms of plaque destabilization (vs. CAD)	Less PE, more CN [36,50,51,52,53]	More CN [23,54]	No dedicated studies	No dedicated studies	No dedicated studies
CAD features in the presence of atherosclerosis on the indicated district	More calcified coronary plaque in the presence of carotid calcifications. Correlations between necrotic core, fibrotic and fibrofatty tissue in carotid and coronary arteries. Association between unstable and vulnerable carotid and coronary plaque features [37,55,56,57,58,59,60,61,62,63,64,65,66]	Greater percent atheroma volume, more coronary calcified plaques, higher vulnerability features (higher lipid burden, macrophage accumulation and CCs) [67,68,69]	More coronary calcification in the presence of SMA calcifications [31]	In CKD patients (higher prevalence in RAS) higher lipid burden and more calcifications in coronary plaques [70,71,72]	More features of coronary plaque vulnerability (more TCFA, lipid rich-plaque, layered plaque, macrophages, plaque rupture) in patients with protruding aortic plaques [73]
Medical therapy	Lifestyle intervention and risk factors control [74,75,76] Antiplatelet therapy is recommended in patients with symptomatic carotid artery stenosis; low-dose aspirin should be considered in asymptomatic carotid stenosis >50% if bleeding risk is low [74] Lipid lowering therapy (statins if tolerated as first step with or without ezetimibe; iPCSK9 showed clinical benefits) [74,77,78,79,80] Colchicine showed efficacy to prevent recurrent strokes [81,82,83]	Lifestyle intervention and risk factors control [74,84,85] Antiplatelet therapy is recommended in patients with symptomatic PAD; low-dose aspirin may be considered in patients with asymptomatic PAD and diabetes mellitus without contraindications; association of aspirin + rivaroxaban should be considered in patients with PAD, high ischemic risk and non-high bleeding risk [74,86,87] Lipid lowering therapy (statins if tolerated as first step with or without ezetimibe; iPCSK9 showed clinical benefits [74,88,89] Colchicine showed efficacy in lower extremity arterial disease; in a preliminary study, canakinumab showed clinical benefits in PAD. Future studies are warranted [90,91]	Lifestyle intervention and risk factors control [74,92] Antiplatelet therapy is recommended after arterial revascularization; lacking evidence about the use of antiplatelet therapy in asymptomatic patients [74] Lipid lowering therapy (statins if tolerated as first step with or without ezetimibe) [74,93]	Lifestyle intervention and risk factors control [74,94] Low-dose aspirin may be considered [74] Lipid lowering therapy (statins if tolerated as first step with or without ezetimibe) [74]	Lifestyle intervention and risk factors control [74] Antiplatelet therapy is recommended in secondary prevention after an embolic event related to aortic atherosclerosis; it should be considered in severe/complex aortic plaques [74] Lipid lowering therapy (statins if tolerated as first step with or without ezetimibe) [74,95]

Abbreviations: CAD = coronary artery disease; CCs = cholesterol crystals; CKD = chronic kidney disease; CN = calcified nodules; FCT = fibrous cap thickness; HDL = high-density lipoprotein; IMA = inferior mesenteric artery; iPCSK9 = Proprotein Convertase Subtilisin/Kexin type 9; IPH = intraplaque hemorrhage; LDL = low-density lipoprotein; PAD = peripheral artery disease; PE = plaque erosion; PIT = pathological intimal thickening; RAS = renal artery stenosis; SMA = superior mesenteric artery; TCFA = thin cap fibroatheroma.

## Abbreviations

AAA	abdominal aortic aneurysms
ACS	acute coronary syndrome
ASCVD	atherosclerotic vascular disease
CAD	coronary artery disease
CACs	coronary artery calcium score
CKD	chronic kidney disease
CTA	computed tomography angiography
IHD	ischemic heart disease
LEAD	lower extremity arterial disease
LRNC	lipid-rich necrotic core
MRI	magnetic resonance imaging
OCT	optical coherence tomography
PAD	peripheral arterial disease
VH-IVUS	virtual histology intravascular ultrasound
